# Magnetic Resonance Evaluation of Multiple Myeloma at 3.0 Tesla: How Do Bone Marrow Plasma Cell Percentage and Selection of Protocols Affect Lesion Conspicuity?

**DOI:** 10.1371/journal.pone.0085931

**Published:** 2014-01-28

**Authors:** Miyuki Takasu, Takayuki Tamura, Yoko Kaichi, Keizo Tanitame, Yuji Akiyama, Shuji Date, Akira Sakai, Yoshiaki Kuroda, Kazuo Awai

**Affiliations:** 1 Department of Diagnostic Radiology, Graduate School of Biomedical Sciences, Hiroshima University, Hiroshima, Japan; 2 Department of Radiation Life Sciences, Fukushima Medical University School of Medicine, Fukushima, Japan; 3 Department of Hematology and Oncology, Research Institute for Radiation Biology and Medicine, Hiroshima University, Hiroshima, Japan; University-Hospital of Parma, Italy

## Abstract

**Purpose:**

To compare various pulse sequences in terms of percent contrast and contrast-to-noise ratio (CNR) for detection of focal multiple myeloma lesions and to assess the dependence of lesion conspicuity on the bone marrow plasma cell percent (BMPC%).

**Materials and Methods:**

Sagittal T_1_-weighted FSE, fat-suppressed T_2_-weighted FSE (FS- T_2_ FSE), fast STIR and iterative decomposition of water and fat with echo asymmetry and least-squares estimation (IDEAL) imaging of the lumbar spine were performed (n = 45). Bone marrow (BM)-focal myeloma lesion percent contrast and CNR were calculated. Spearman rank correlation coefficients were obtained between percent contrast, CNR and BMPC%. Percent contrasts and CNRs were compared among the three imaging sequences.

**Results:**

BM-focal lesion percent contrasts, CNRs and BMPC% showed significant negative correlations in the three fat-suppression techniques. Percent contrast and CNRs were significantly higher for FS- T2 FSE than for STIR (*P*<0.01, *P*<0.05, respectively), but no significant differences were found among the three fat-suppression methods in the low tumor load BM group.

**Conclusion:**

The higher BMPC% was within BM, the less conspicuous the focal lesion was on fat-suppressed MRI. The most effective protocol for detecting focal lesions was FS- T_2_ FSE. In the high tumor load BM group, no significant differences in lesion conspicuity were identified among the three fat-suppression techniques.

## Introduction

Multiple myeloma is a plasma-cell malignancy characterized by the presence of lytic bone disease causing severe bone pain, pathological fractures, spinal cord compression and hypercalcemia [1]. Up to 90% of myeloma patients develop osteolytic lesions during the course of the disease [2]. These lesions occur predominantly in the axial skeleton (i.e., skull, spine, rib cage and pelvis), as well as the proximal areas of the arms and legs [3].

In 2003, the International Myeloma Working Group introduced the Durie-Salmon PLUS staging system [4], which takes into account the number of lesions detected by magnetic resonance imaging (MRI) or ^18^F-fluorodeoxyglucose positron emission tomography (PET). In patients with active myeloma, the number of lesions on MRI correlates very well with treatment outcomes and overall survival [5]. This excellent correlation with survival outcome is the primary reason for the inclusion of MRI into the Durie-Salmon PLUS system. However, counting focal lesions can be somewhat confusing, with variegated or diffuse patterns of tumor cell infiltration reportedly found in 57% of cases on T_1_-weighted imaging [6], obstructing detection of focal lesions. Furthermore, the Durie-Salmon PLUS staging system does not include the presence or absence of diffuse infiltration of tumor cells into the bone marrow (BM). This means that focal myeloma lesions must be detected regardless of any abnormality in background BM. The optimal MRI sequence for detecting focal bone lesions thus remains to be determined.

Various MR pulse sequences are available for evaluating the spine, including fast spin-echo (FSE) imaging with and without fat suppression, short inversion time inversion recovery (STIR) imaging, and the iterative decomposition of water and fat with echo asymmetry and least-squares estimation (IDEAL) technique. An important feature to detect focal lesions in the spine is suppression of marrow fat, because subtle high-intensity lesions can be obscured by the high signals from marrow fat on routine T_2_-weighted spin-echo imaging.

The STIR pulse sequence offers high sensitivity for detecting neoplasia due to its ability to show the combined effects of prolonged T_1_ and T_2_ relaxation times of these pathological tissues [7]–[9]. In 2000, Nakatsu et al. [9] reported STIR as superior to T_2_-weighted FSE with fat saturation for detection of metastatic lesions, in terms of lesion conspicuity.

A newer approach for achieving fat suppression is the simple spectroscopic imaging technique, from which IDEAL was later developed, originally published by Dixon [10]. In the original implementation, Dixon acquired an image with water and fat signals in-phase and another image with water and fat signals 180° out-of-phase. Dixon showed that simple summation and subtraction of the two images yield a water-only image and a fat-only image, respectively. The most serious problem for the Dixon techniques is B_0_ inhomogeneity, which appears as phase errors in the acquired Dixon images. Without proper phase correction, the simple summation and subtraction approach results in incomplete water and fat separation, thus making the Dixon techniques also sensitive to magnetic field inhomogeneity. After Dixon's original work, Yeung and Kormos [11], Glover and Schneider [12], and Glover [13] showed that phase errors can actually be determined by acquiring an additional image. This analytical method for water and fat separation involves acquisition of three separate images with different water and fat relative phase angles and determination of water and fat on a per-pixel basis through an iterative least-squares process [14]. Integration of the region-growing and iterative linear least-squares methods has improved water-fat separation compared with using the original iterative process alone [15]. IDEAL is one of the three-point water-fat separation methods that uses asymmetric echoes and least-squares fitting to achieve the maximum possible signal-to-noise ratio (SNR) on MRI [14], [16]–[18]. Calculation of the quantity of magnetic inhomogeneity in each pixel from data using the least-squares method was employed to generate the field map of IDEAL. By correcting phase shift in each pixel using this field map, robust fat suppression can be achieved, resulting in more accurate and confident interpretations in areas of B_0_ inhomogeneity.

The present study was performed to compare lesion conspicuity between T_1_-weighted fast spin echo (T_1_ FSE), fat-suppressed T_2_-weighted FSE (FS- T_2_ FSE), STIR, and T_2_-weighted FSE IDEAL sequences in terms of percent contrast and contrast-to-noise ratio (CNR) between focal myeloma lesion and background BM, and to assess the dependence of lesion conspicuity on myeloma cell mass in background BM in patients with multiple myeloma.

## Materials and Methods

### Study participants

All study protocols were approved by the appropriate institutional review boards (University Hospital Medical Information Network Clinical Trials Registry [UMIN– 112 CTR] number, UMIN000003663). Each participant provided written informed consent before undergoing MRI.

Spinal MRI was performed in 45 patients with multiple myeloma between June 2010 and November 2013. The criteria used for diagnosis were taken from the classification of Durie and Salmon [18]. Diagnoses were confirmed when there were more than 10% clonal plasma cells in BM samples of the iliac crest. We excluded patients who had undergone chemo- or radiotherapy. Patients comprised 25 men (mean age, 65.2 years; range, 52–81 years) and 20 women (mean age, 67.3 years; range, 55–78 years). Of the 45 patients in the staging cohort, 8 had asymptomatic myeloma (Durie-Salmon stage I), and 37 had symptomatic myeloma (Durie-Salmon stage II, n = 21; Durie-Salmon stage III, n = 16). The distinction between symptomatic and asymptomatic myeloma depended on the presence or absence of myeloma-related organ dysfunction according to the criteria of the International Myeloma Working Group [18]. The 37 symptomatic patients had symptoms described in the CRAB criteria (renal insufficiency, n = 9; anemia, n = 24; focal lytic bone lesions, n = 37). Two authors (with 20 years of expertise in spinal imaging and 12 years of expertise in hematology) reviewed all medical and clinical records to collect all available data.

### BM examination

We estimated the percent of BM plasma cells (BMPC%) in BM biopsy specimens obtained from the iliac crest.

### Spinal MRI and quantitative study

Imaging was performed using a 3.0-T MRI unit (Signa HDxt 3T; GE Healthcare Milwaukee, WI) and the following sequences (Table 1): sagittal T_1_ FSE; sagittal FS- T_2_ FSE (with a chemical shift selective (CHESS) technique); sagittal fast STIR imaging; and a sagittal IDEAL T_2_ FSE sequence. Co-registered water, fat, in-phase (water+fat) and out-of-phase (water-fat) images were generated by the IDEAL software.

**Table 1:** Acquisition parameters for MR sequences.

Sequence	TR/TE/TI (ms)	NEX	FOV (mm)	Matrix	Slice thickness (mm)	Bandwidth (kHz)	Imaging time (min:s)
Water image of IDEAL	4000/112.4	6	300	384×192	4	83.3	5:12
Fat-suppressed T_2_ FSE	4000/116	2	300	320×288	4	62.5	2:16
Fast STIR	4200/106.8/190	2	300	320×224	4	31.2	3:01
T_1_ FSE	700/11.8	2	300	512×224	4	41.7	2:38

NEX, number of signal averages.

Mean signal intensity and standard deviation were calculated by placing operator-determined regions of interest (ROIs) within the focal myeloma lesions and within BM without focal lesions. The ROI for BM was defined manually within the internal part of the L1–L3 vertebral bodies in the midsagittal images, as described elsewhere [19], which did not include focal myeloma lesions, because these spinal levels were less affected by degenerative disc disease compared to lower lumbar elements. Signal intensity values for BM were then calculated as the mean value obtained from the three vertebral bodies and used as background BM. The ROI was placed at the same location on all sequences. Each ROI had an area of 254–519 mm^2^ for BM and 58–222 mm^2^ for myeloma lesion. Focal myeloma lesion was defined as an area of low signal intensity on T_1_-weighted images and high or intermediate signal intensity on in-phase images of IDEAL with a relatively well-defined margin larger than 0.7 cm in the long axis. All focal myeloma lesions were confirmed to be shown as lytic lesions on computed tomography (CT). The largest focal lesion in each patient was measured. For each MRI examination, the percent contrast and CNR between BM and focal myeloma lesion (n = 45) was measured using the following equations:
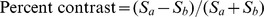


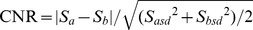
where S_a_ and S_b_ are mean signal intensity and S_asd_ and S_bsd_ are the standard deviation of intensities of focal myeloma lesion and BM, respectively.

### Statistical analysis

All statistical analyses were performed using statistical software (Excel 2007; Microsoft, Redmond, WA).

Spearman rank correlation coefficients (ρ) were calculated to investigate correlations between percent contrast, CNR and BMPC%. One-way analysis of variance with Scheffé's post hoc test was used to compare percent contrast and CNR among the four different groups (i.e., T_1_ FSE, FS- T_2_ FSE, fast STIR, and water image of IDEAL) for 45 patients with focal myeloma lesions. Differences were considered significant at the *P*<0.05.

## Results

**Figure 1:**
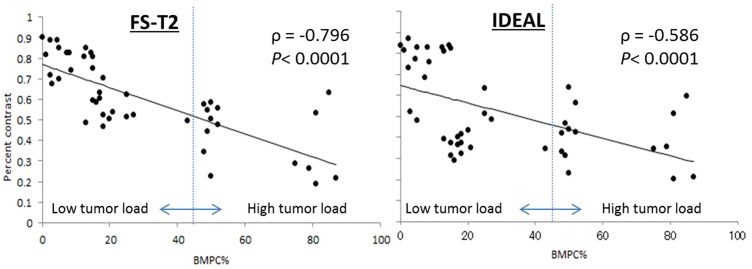
Graph of the BMPC% versus percent contrast. The linear regression curve is shown. Vertical line corresponds to a BMPC% of 45%.

A significant correlation was seen between percent contrast and BMPC%, with Spearman correlation coefficients of −0.586 (*P*<0.0001), −0.796 (*P*<0.0001), and −0.494 (*P*<0.01) for water image of IDEAL, FS- T_2_ FSE, and STIR, respectively (Table 2, Fig. 1). Between CNR and BMPC%, there were also significant correlations with Spearman correlation coefficients of −0.664 (*P*<0.0001), −0.709 (*P*<0.0001), and −0.500 (*P*<0.001), for water image of IDEAL, FS- T_2_ FSE, and STIR, respectively (Table 2). No significant correlation was evident between percent contrast and CNR with BMPC% for T_1_ FSE. Negative correlations between percent contrast and CNR with BMPC% for FS- T_2_ FSE were stronger than those for water image of IDEAL. Correlations between percent contrast and CNR with BMPC% for STIR were the weakest among the three fat-suppression techniques.

**Table 2:** Results of Spearman rank correlation for percent contrast and CNR with BMPC% among the four different sequences in multiple myeloma (n = 45).

	Percent contrast		CNR	
Sequence	ρ	*p*	ρ	*p*
IDEAL	−0.580	<0.0001	−0.664	<0.0001
FS-T_2_ FSE	−0.796	<0.0001	−0.709	<0.0001
Fast STIR	−0.494	<0.001	0.500	<0.001
T_1_ FSE	−0.086	0.57	−0.100	0.48

IDEAL, water image of IDEAL; FS-T_2_ FSE, fat-suppressed T_2_ FSE.

This result means that the higher the BMPC%, the less conspicuous the focal lesion is on fat-suppressed MRI. Therefore, in light of the difficulty of detecting focal lesions in low fat-containing marrow, i.e., a high tumor load BM, we categorized patients into two groups: a high tumor load BM group (n = 15), with BMPC% ≥45%; and a low tumor load BM group (n = 30), with BMPC%<45%.

**Figure 2:**
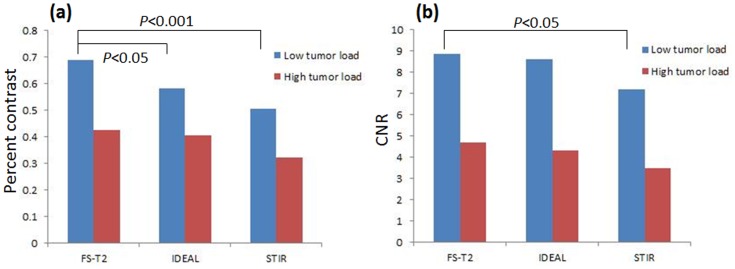
Percent contrast and CNR comparison among the three different fat-suppression sequences. BM-focal lesion percent contrast (a) and CNR (b) are significantly greater for FS-T2 FSE than for STIR in the low tumor load BM groups (P<0.001, P<0.05, respectively). In the low tumor load BM group, percent contrast is significantly higher for FS-T2 FSE than for water image of IDEAL (p<0.05).

BM-focal lesion percent contrast and CNR were significantly greater for FS- T_2_ FSE than for STIR in the low tumor load BM group (*P*<0.001, *P*<0.05, respectively, Table 3, Fig. 2). In the low tumor load BM group, percent contrast was significantly higher for FS-T2 FSE than for water image of IDEAL (*P*<0.05), but this was not the case in the comparison of CNR. No significant difference was found among the three fat-suppression methods in the high tumor load BM group, although mean values of BM-focal lesion percent contrast and CNR were highest for FS- T2 FSE and lowest for STIR.

**Table 3:** Comparison of percent contrast and CNR among the three fat-suppression sequences in multiple myeloma.

	IDEAL	FS-T2 FSE	STIR
Percent contrast			
Total (n = 45)	0.52±0.21	0.60±0.19††	0.44±0.22
Low tumor load BM* (n = 30)	0.58±0.21	0.69±0.14+ ^, ^ ††	0.51±0.24
High tumor load BM** (n = 15)	0.40±0.14	0.43±0.15†	0.32±0.10
CNR			
Total (n = 45)	7.17±3.70	7.47±3.74†	5.97±4.24
Low tumor load BM* (n = 30)	8.60±3.61	8.87±3.7†	7.21±4.6
High tumor load BM** (n = 15)	4.32±1.44	4.68±1.49†	3.50±1.20

IDEAL, water image of IDEAL; FS-T_2_ FSE, fat-suppressed T_2_ FSE; BM, bone marrow.

Data are shown as mean ± standard deviation.

* Low tumor load BM: bone marrow with fat-signal fraction <45%.

** High tumor load BM: bone marrow with fat-signal fraction ≥45%.

+ *P*<0.05, Fat-suppressed T_2_ FSE vs. water image of IDEAL.

†† *P*<0.01,

† *P*<0.05, Fat-suppressed T_2_ FSE vs. fast STIR.

**Figure 3:**
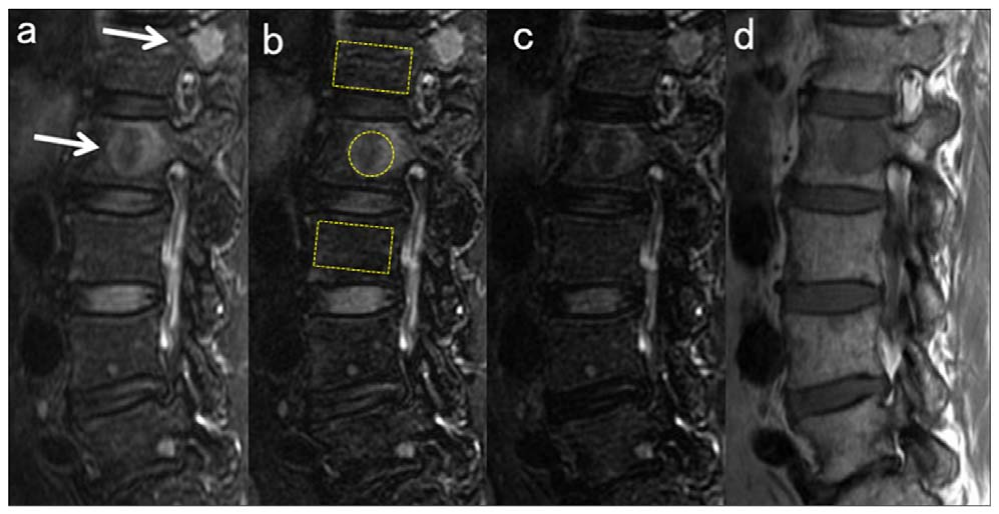
Sagittal MRI in a 58-year-old woman with focal myeloma lesions in the low tumor load group (BMPC%, 12%). **a**) Water image of IDEAL; **b**) FS- T_2_ FSE; **c**) STIR; and **d**) T_1_ FSE. ROIs of lesion and background BM used for the percent contrast computation are demarcated in the images. Actual ROIs of BM are placed in similar locations in the midsagittal images. The focal myeloma lesions in L1 and L2 are easily detected as a high intensity signal on fat-suppressed images (white arrows). Among the three fat-suppressed images, signal hyperintensity of the lesion is less conspicuous in STIR (c) than in water image of IDEAL (a), which seems to be due to the lower SNR in STIR.

**Figure 4:**
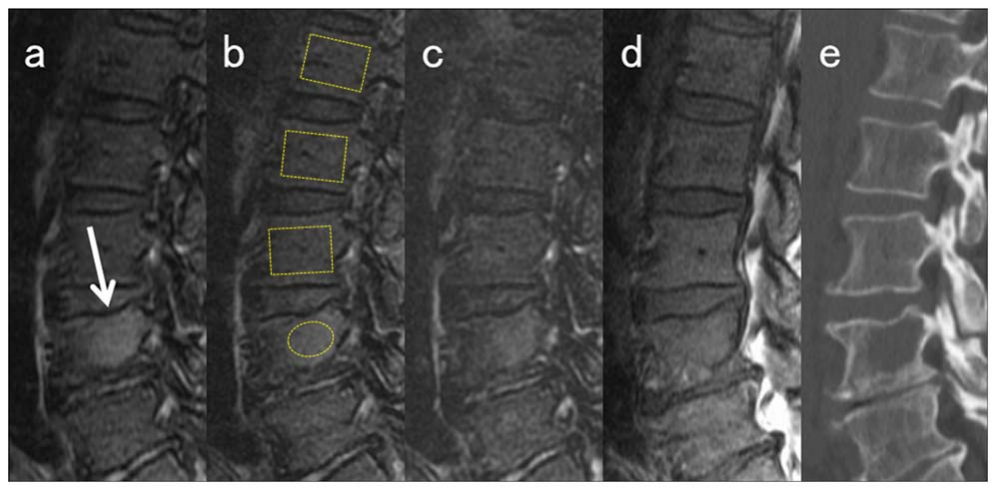
Sagittal MRI in a 65-year-old man with combined focal and diffuse infiltration pattern (BMPC%, 71%). **a**) Water image of IDEAL; **b**) FS-T_2_ FSE; **c**) STIR; **d**) T_1_ FSE; and **e**) CT. ROIs of lesion and background BM used for the percent contrast computation are demarcated in the images. The actual ROIs of BM are placed in similar locations in the midsagittal images. The focal myeloma lesion in L4 is seen as a slightly high intensity signal on fat-suppressed images (white arrows) but is less conspicuous compared to the lesion in Figure 3. Among the three fat-suppressed images, signal hyperintensity of the lesion is less conspicuous in STIR (c) than in water image of IDEAL (a) or FS- T_2_ FSE (b). The focal lesion is not detected on T_1_ FSE (d).

Representative images are shown in Figures 3 and 4.

## Discussion

The presence of focal lesions on MRI has been correlated with shorter overall survival in several studies of patients with multiple myeloma [5], [20]. Since the Durie-Salmon PLUS staging system does not mention about diffuse infiltration pattern of tumor cells in the BM, focal myeloma lesions must be detected regardless of any diffuse abnormality in background BM. In this study, BM-focal lesion percent contrasts and BMPC% showed significant negative correlations in the three fat suppression techniques. This means that the higher the BMPC% within BM, the less conspicuous the focal lesion is on fat-suppressed MRI. We attributed the lower percent contrast on fat-suppressed images with higher BMPC% to increased signal intensity of background BM, mainly caused by T_2_ prolongation by diffusely infiltrated myeloma cells, which can reduce the signal intensity contrast between focal lesion and background BM. Furthermore, results of post hoc tests according to groups categorized by tumor load also demonstrated no significant difference among the three fat-suppression methods in the high tumor load BM group. This study did not identify any clearly superior fat-suppression technique for detecting focal myeloma lesions in the high tumor load BM. In clinical settings, other modalities such as CT with multiplanar reconstruction and PET/CT might be helpful to detect focal lesions.

BM-focal lesion percent contrast and CNR for FS- T_2_ FSE were significantly higher than STIR in the analyses for total lesions and for lesions in the low tumor load BM group. This finding can be explained by the improvement of the saturation pulse of CHESS technique in 3-T MRI. In 2011, Tagliafico et al. [21] compared 1.5- and 3-T MRI of the brachial plexus and demonstrated that CNR in 3-T MRI was significantly better in FS-T2 FSE sequences. On the other hand, Sormaala et al. [22] found no noteworthy differences in the sensitivity of 1.5- and 3-T images when they evaluated bone edema caused by acute bone stress in the foot using STIR. In addition, when imaging at 3 T, the longer T1 of tissue at higher field strengths would decrease lesion contrast if imaging time was the same as that at 1.5 T. Furthermore, even though STIR is insensitive to B_0_ inhomogeneities, it may be sensitive to B_1_ inhomogeneities, particularly at higher field strengths, where B_1_ may be more inhomogeneous. In addition, chemical shift is larger in 3 T than in 1.5 T. These facts may partly explain the opposite result in this study regarding the percent contrast of FS- FSE T2 and STIR.

The present study showed that the percent contrast for FS- T2 FSE was significantly greater than that of water image of IDEAL in the low tumor load group. The exact cause is unclear, but may be related to the inherent homogeneity of magnetic fields in the lumbar spine area. In 2004, Ma et al. [23] reported that the fast three-point water-fat separation technique provides superior fat suppression and lesion conspicuity in the spine by 1.5 T, and can potentially be used as an alternative to T2-weighted imaging of the spine. They analyzed the whole spinal column, including the cervical and thoracic spine, as often being subject to susceptibility artifacts around the shoulder, mediastinum, or diaphragm. On the other hand, we investigated contrast characteristics focused on the lumbar spine, where fat suppression was fairly uniform over a field of view, which reduced the superiority of IDEAL for obtaining fat-suppressed images.

Our protocol for assessment of bone lesion for multiple myeloma did not include diffusion-weighted imaging. In 2011, Sommer et al. reported the diagnostic potential of diffusion-weighted imaging with background suppression (DWIBS) in the detection of focal BM lesions from multiple myeloma [24]. According to that report, the CNR values provided by DWIBS in patients with high serum concentration of M-component are slightly higher than those of T2-weighted STIR. Since they did not perform CNR analysis for IDEAL or FS- T2 FSE, whether spinal diffusion-weighted imaging could yield better performance in delineation of focal myeloma lesions compared to IDEAL and FS- T2 FSE cannot be determined. However, diffusion-weighted images suffer from susceptibility artifacts and image distortion caused by eddy currents and cannot yet achieve high spatial resolution. Since the Durie-Salmon PLUS staging system does not mention lesion size, we believe that MRI with reasonable spatial resolution and without significant degradation is needed for the purpose of counting focal lesions.

Several limitations to this study must be considered when interpreting the present findings. First, the scanning parameters used for fat-suppression techniques were not identical, to adapt acquisition time to clinical practice. FS- T_2_ FSE used twice the bandwidth of STIR, presenting a more favorable condition for STIR regarding SNR. IDEAL used three times the NEX of the other two sequences, presenting a more favorable condition for IDEAL. We therefore consider that these differences in imaging parameters are unlikely to have had any substantial effect on our results. Second, biopsy specimens of spinal BM were not obtained in this study; instead, we calculated BMPC% from BM samples of the iliac crest. Neoplastic plasma cells tend to form clusters, which may be small or large [25]. Variability of the histopathological pattern and spatial distribution for multiple myeloma could have resulted in some error in this study. Other factors with possible differences in spatial distribution, including hematopoietic BM and degenerative disc disease, might thus have influenced the fat fraction of BM, resulting in altered signal intensity in fat-suppressed MRI. The normal distribution of hematopoietic BM in the adult, in which only the axial skeleton and proximal shafts of the femurs and humeri contain hematopoietic marrow, is completed by around 25 years old. With advancing age, generally beyond 40 years old, the vertebral BM becomes increasingly replaced with fatty marrow [26]. The BM also undergoes changes due to dietary changes, anemia, chronic hypoxia, chemotherapy, and other medications, through the actions of various cytokines [27], [28]. Such interindividual variability of fatty marrow replacement could thus have affected our results. We also acknowledge that MRI in our study was limited to the lumbar vertebrae. The use of specific vertebral bodies might not be appropriate for assessing lesion conspicuity of focal myeloma lesions, which can occur in any bones susceptible to magnetic field inhomogeneity. Further studies on lesion conspicuity on whole-body MRI combined with information on the amount of myeloma cells are warranted.

In conclusion, the higher the BMPC% obtained from biopsy, the less conspicuous the focal lesion on fat-suppressed MRI. To the best of our knowledge, this dependence of lesion conspicuity on myeloma mass in the BM has not been described previously, and could have clinical implications for staging and treatment planning in cases of multiple myeloma. The most effective protocol for detecting focal lesions was FS- T_2_ FSE. No significant differences in lesion conspicuity were found among fat suppression techniques in the high tumor load BM group.
